# Discovery and Development Strategies for SARS-CoV-2 NSP3 Macrodomain Inhibitors

**DOI:** 10.3390/pathogens12020324

**Published:** 2023-02-15

**Authors:** Marion Schuller, Tryfon Zarganes-Tzitzikas, James Bennett, Stephane De Cesco, Daren Fearon, Frank von Delft, Oleg Fedorov, Paul E. Brennan, Ivan Ahel

**Affiliations:** 1 Sir William Dunn School of Pathology, University of Oxford, Oxford OX1 3RE, UK; 2 Centre for Medicines Discovery, University of Oxford, Headington OX3 7DQ, UK; 3 Diamond Light Source Ltd., Harwell Science and Innovation Campus, Didcot OX11 0DE, UK; 4 Research Complex at Harwell, Harwell Science and Innovation Campus, Didcot OX11 0FA, UK; 5 Structural Genomics Consortium, University of Oxford, Headington OX3 7DQ, UK; 6 Department of Biochemistry, University of Johannesburg, Auckland Park, Johannesburg 2006, South Africa

**Keywords:** ADP-ribosylation, macrodomain, SARS-CoV-2, COVID-19, non-structural protein 3 (NSP3), drug discovery and development, virtual screening

## Abstract

The worldwide public health and socioeconomic consequences caused by the COVID-19 pandemic highlight the importance of increasing preparedness for viral disease outbreaks by providing rapid disease prevention and treatment strategies. The NSP3 macrodomain of coronaviruses including SARS-CoV-2 is among the viral protein repertoire that was identified as a potential target for the development of antiviral agents, due to its critical role in viral replication and consequent pathogenicity in the host. By combining virtual and biophysical screening efforts, we discovered several experimental small molecules and FDA-approved drugs as inhibitors of the NSP3 macrodomain. Analogue characterisation of the hit matter and crystallographic studies confirming binding modes, including that of the antibiotic compound aztreonam, to the active site of the macrodomain provide valuable structure–activity relationship information that support current approaches and open up new avenues for NSP3 macrodomain inhibitor development.

## 1. Introduction

The outbreak of coronavirus disease 2019 (COVID-19) caused by severe acute respiratory syndrome coronavirus 2 (SARS-CoV-2) has become a major public health challenge over the last two years, claiming over 6 million lives so far while being accompanied by severe socioeconomic consequences worldwide [1,2,3]. The impact of this recent pandemic together with previous coronaviral outbreaks within the past two decades, including SARS-CoV in 2002–2003 and MERS-CoV in 2012–2015 [4], underlines the importance of developing strategies for effectively gaining control of such and general viral disease outbreaks. Apart from non-pharmacologic interventions and prevention measures achievable by vaccines, the development of antiviral agents presents an alternative to increase preparedness by providing rapid disease treatment possibilities. 

SARS-CoV-2 is characterised as an enveloped single-stranded positive sense RNA β-coronavirus whose genome encodes for 29 proteins essential for the viral life cycle and its modulation of host immune responses [5,6,7]. Proteins involved in the viral replication machinery are thereby in particular focus as drug targets. Thus, the RNA polymerase is targeted with nucleoside analogues (e.g., remdesivir or molnupiravir) to inhibit the genome replication and gene transcription of SARS-CoV-2 [8,9]. Moreover, the viral proteases, the main protease (M^pro^), and the papain-like protease 2 (PL2^pro^), are inhibited by peptide analogues (e.g., nirmatrelvir) and small molecules to prevent the processing of two polypeptides into constituent viral non-structural proteins (NSP) required for viral replication [9,10,11]. NSP3 is thereby the largest multi-domain protein produced by coronaviruses and is itself an essential component of the replication and transcription complex [12]. SARS-CoV-2 NSP3 features eight (out of 15) domains that exist in all known coronaviruses, including ubiquitin-like domains, PL2^pro^, transmembrane regions, and a macrodomain (Mac1) [12]. Macrodomains are highly conserved domains found in all kingdoms of life [13] and recognise ADP-ribosylation modifications on proteins and nucleic acids catalysed by poly(ADP-ribosyl)polymerases (PARPs) [14,15]. The interferon (IFN) response triggered through viral infections thereby induces the gene expression of several PARP family members, i.e., PARP7 and PARPs 9–14, whose ADP-ribosylation signalling activity establishes an antiviral environment [16,17]. While, for instance, the antiviral effect of PARP12 was shown to be achieved at least partially through the inhibition of protein translation and by promoting the ADP-ribosylation-dependent degradation of viral proteins [16,18], PARP9 provides a possibility of viral infection control in complex with DTX3L by targeting EMCV 3C protease for ubiquitination and degradation [19]. Furthermore, PARP14 was shown to promote anti-inflammatory interleukin-4-mediated signalling pathways by activating STAT6-dependent gene expression and inhibiting STAT-1-dependent gene expression [20,21]. However, PARP14 expression is also induced by interferon (IFN), and it enhances host IFN responses to lipopolysaccharide (LPS), poly(I:C), and viral infection, indicating a role for PARP14 in restricting viral and bacterial infections [22,23,24]. However, viral macrodomains such as the SARS-CoV-2 macrodomain evolved with ADP-ribosyl hydrolase activity to reverse PARP-catalysed ADP-ribosylation, thus providing the virus with a strategy to counteract these host defence mechanisms [25,26]. Studies in mice confirmed that mutations of the SARS-CoV macrodomain impairing its catalytic activity led to virus attenuation, a reduction in viral loads, and a stronger immune response following infection compared to the wild-type virus, thereby rendering the virus nonlethal [23,27]. Thus, with the NSP3 macrodomain being critical for replication and pathogenicity in the host for coronaviruses, and similarly for alphaviruses and Hepatitis E virus [28], the macrodomain was established as a therapeutic target for SARS-CoV-2 infection [26,29].

Although the molecular physiological substrates and exact mechanisms of the enzymatic ‘arms race’ between antiviral PARP and coronaviral macrodomain are still unclear, the macrodomain itself has been in intense focus to pave the way for the development of a new antiviral drug. Its well-defined binding pocket along with its high amenability for structural and biochemical characterisation fostered rapid assay development for in vitro compound screening and discovery [30,31,32,33], allowed the elucidation of its catalytic mechanism [34], and gave insights into druggability and plasticity by crystallographic [35,36], NMR [37], and computational molecular dynamics [38,39] approaches. Furthermore, due to the general conservation of the macrodomain fold, the screening of focused chemical libraries curated from inhibitor development programmes of the PARG macrodomain could be performed to further support SARS-CoV-2 NSP3 macrodomain drug development [40]. 

In this study, we present our approaches to contribute to the initial drug discovery phase for the SARS-CoV-2 NSP3 macrodomain, also referred to as ‘Mac1’. We performed computational docking studies which provided insights into the chemical matter to be considered for targeting the active site of the macrodomain. Using an established HTRF-based screening assay for NSP3 Mac1, we furthermore screened medium-sized libraries comprising either experimental small molecules or FDA-approved drugs. The former library screening approach enabled the identification of four molecular scaffolds with inhibitory effects on NSP3 Mac1, whereby initial structure–activity relations were obtained by analogue characterisation. Moreover, we discovered with our FDA-approved library screening that the active site of the SARS-CoV-2 macrodomain can be inhibited by antibiotic agents including aztreonam, whose binding we confirmed by crystallographic studies. Altogether, our studies provide valuable chemical starting points for future inhibitor development for the NSP3 macrodomain. 

## 2. Methods

### 2.1. Materials, Reagents, and Chemicals

Crystallisation screens were procured from Hampton Research. The ADPr-peptide with sequence ARTK(Bio)QTARK(Aoa-RADP)S used for HTRF assays was purchased from Cambridge Peptides. All remaining chemicals were purchased from Sigma unless stated otherwise. The BioAscent library of 125,000 compounds was purchased from BioAscent (https://www.bioascent.com/integrated-drug-discovery/in-house-diversity-and-fragment-libraries, accessed on 24 April 2020). The MIDAS library was a generous gift from Allan Jordan of Cancer Research UK. 

### 2.2. Constructs

SARS-CoV-2 NSP3 Mac1 (residues 206–379) cloned into a pDEST17 vector with N-terminal His_6_-tag was used for performing HTRF assays [26]. SARS-CoV-2 NSP3 Mac1 (residues 207–373) cloned into a pNIC28-Bsa4 expression vector with N-terminal His_6_-TEV cleavage site [36] was used for protein crystallisation. 

### 2.3. Protein Expression and Purification for Crystallisation

*E. coli* Rosetta strain BL21(DE3) was transformed with the constructs encoding SARS-CoV-2 NSP3 macrodomains and grown at 37°C in Terrific Broth (Merck Millipore, Burlington, MA, US), which was supplemented with 50 μg/mL of kanamycin and 35 μg/mL of chloramphenicol. After reaching an OD_600nm_ of 1.0–1.2, the temperature was lowered to 18°C prior to the induction of protein expression overnight (O/N) by adding 0.5 mM of IPTG. The harvested cells were resuspended in lysis buffer (50 mM of HEPES (pH 7.4), 500 mM of NaCl, 5% glycerol, 20 mM of imidazole, 0.5 mM of TCEP, cOmplete EDTA-free protease inhibitors (Roche, Basel, Switzerland) and stored at −20°C until purification. 

For protein purification, pellets were gently thawed and lysed by high-pressure homogenisation. DNA was digested using Benzonase Nuclease (Merck Life Science, Darmstadt, Germany). Proteins were purified by immobilised metal affinity chromatography (IMAC) using Ni-Sepharose resin (GE Healthcare, Chicago, IL, US) and eluted stepwise in binding buffer containing 40–500 mM imidazole. Typically, a high salt wash with 1 M of NaCl was combined with the first elution step including 40 mM of imidazole. Protein purified for performing HTRF assays was further purified by size exclusion chromatography (SEC) (Superdex 75, GE Healthcare) in a buffer consisting of 25 mM of HEPES (pH 7.5), 300 mM of NaCl, 5% glycerol, and 0.5 mM of TCEP. For protein purified for the crystallisation experiments, the removal of the hexahistidine tag was carried out after the first Ni-IMAC step by the addition of recombinant TEV protease during O/N dialysis into buffer without imidazole, followed by purification on a second IMAC column. Finally, protein was purified by SEC (Superdex 75, GE Healthcare) in a buffer consisting of 20 mM of HEPES (pH 8.0), 250 mM of NaCl and 2 mM of DTT. The proteins were characterised by SDS-PAGE, then flash-frozen in liquid nitrogen and stored at −80°C until required.

### 2.4. HTRF Assay

The inhibition of SARS-CoV-2 NSP3 Mac1 was assessed by the displacement of an ADP-ribose-conjugated biotin peptide from His_6_-tagged protein using an HTRF-technology-based screening assay, which was performed as previously described [36]. Compound library screens (including the MIDAS and FDA-approved screening set and the curated BioAscent hit compound library) were performed at a compound concentration of 25 µM in duplicate measurements, while for hit confirmation, IC_50_ curves were acquired with a top compound concentration of 125 µM (MIDAS and FDA-approved hit compounds) or 187 µM (BioAscent hit compounds), followed by an 8-point 1:1 dilution series in duplicate measurements. The compounds were dispensed into ProxiPlate-384 Plus (PerkinElmer, Waltham, MA, US) assay plates using an Echo 525 liquid handler (Labcyte, San Jose, CA, US). Binding assays were conducted in a final volume of 16 μL with 12.5 nM of SARS-CoV-2 NSP3 Mac1, 400 nM of peptide ARTK(Bio)QTARK(Aoa-RADP)S, 1:20,000 Anti-His_6_-Eu^3+^ cryptate (HTRF donor, PerkinElmer), and 1:125 Streptavidin-XL665 (HTRF acceptor, PerkinElmer) in assay buffer (25 mM of HEPES pH 7.0, 20 mM of NaCl, 0.05% bovine serum albumin and 0.05% Tween-20). Assay reagents were dispensed into plates using a Multidrop combi (Thermo Scientific, Waltham, MA, US). Macrodomain protein and peptide were first dispensed and incubated for 30 min at room temperature. This was followed by the addition of the HTRF reagents and incubation at room temperature for 1 h. Fluorescence was measured using a PHERAstar microplate reader (BMG) using the HTRF module with dual emission protocol (A = excitation of 320 nm, emission of 665 nm, and B = excitation of 320 nm, emission of 620 nm). Raw data were processed to give an HTRF ratio (channel A/B × 10,000), which was used to generate IC_50_ curves. The IC_50_ values were determined by nonlinear regression using GraphPad Prism v.9 (GraphPad Software, San Diego, CA, USA). Of note is that we judged—based on our experience in medicinal chemistry and FRET-based assays, as well as references in the literature (e.g., Baell and Walters, 2014 [41])—the screening compounds for the presence of chemical features known to cause assay interference and promiscuous binding behaviour. Compounds were excluded from the hit validation processes without further biophysical testing or computational predictions when certain motifs were identified. However, where stated as “showed assay effects at higher concentrations”, the compounds did not show structural features suspicious for assay interference per se and were tested in the HTRF-based assay. At higher compound concentrations of the titration, we observed a decrease or were even unable to determine Mac1 inhibition values, indicating that these compounds had unspecific assay effects unrelated to true Mac1 inhibition. 

### 2.5. Crystallisation, Crystal Soaking, and Data Processing

The purified SARS-CoV-2 NSP3 Mac1 protein was concentrated to 47 mg/mL, and crystallisation drops were set-up in MRC two-well crystallization microplates (Swissci, Buckinghamshire, UK) using the Mosquito Crystal robot (TTP Labtech, Cambridgeshire, UK) with protein to reservoir ratios of 1:1 and 1:2, in a 150 nl total volume equilibrated against 75 µL of reservoir solution containing 100 mM CHES pH 9.5 and 30% PEG3000. To ease crystallisation for soaking experiments, ~5 crystals were harvested and prepared as seed stock using a Seed Bead Kit (Hampton Research, Aliso Viejo, CA, US) in 100 nl of reservoir solution. An amount of 20 nl of a 1:500 dilution of the resulting seed stock was added to the crystallisation experiments. The compounds were soaked into crystals by adding 0.5 µL of dissolved compounds directly to the crystallisation drops. After incubation for 1–3 h, the crystals were harvested using reservoir solution supplemented with 20% ethylene glycol (*v*/*v*) as a cryo-protectant prior to flash freezing in liquid nitrogen. X-ray data were collected at beamline I03 at Diamond Light Source (Rutherford Appleton Laboratory, Harwell, UK) and data collection statistics are given in Supplementary Table S1. 

The X-ray data were processed using the XIA2-DIALS platform [42], and phase information was obtained using the molecular replacement method with PHASER [43] using 7KQP as template. Atomic models were improved following consecutive cycles of manual building in COOT [44] and structure refinement in REFMAC5 [45]. The structures were refined to good Ramachandran statistics, and MolProbity [46] was used to validate the models prior to deposition in the PDB. The processing and refinement statistics are given in Supplementary Table S1. Structural alignments and analyses, as well as figure preparation, were carried out using PyMol (Molecular Graphics System, Version 2.3.3 Schrӧdinger, LLC., New York, NY, USA). 

### 2.6. Virtual Screening

The structure of the NSP3 macrodomain (‘Mac1’) was downloaded from the Protein Data Bank (rcsb.org) as a PDB file (6W02). The macrodomain displayed a closed conformation. All water molecules were removed, except four in the binding site that formed water-mediated hydrogen bonds between ADP-ribose and the protein (wb32, wb60, wb71, and wb107). The protein was prepared for docking in Schrodinger. The BioAscent library was prepared for docking using Schrodinger ligprep with racemic compounds being expanded to include discreet enantiomers. The compounds were docked into the protein using Glide SP with default parameters and the top scoring enantiomer kept for evaluation. The top 2000 highest scoring compounds were selected for IC_50_ determination, of which 1786 compounds could be supplied by the company for biophysical characterisation in the HTRF assay. ChemDraw 21.0.0 was used for the visualization and drawing of compound structures.

## 3. Results

### 3.1. NSP3 Macrodomain Virtual Ligand Screen

**Figure 1:**
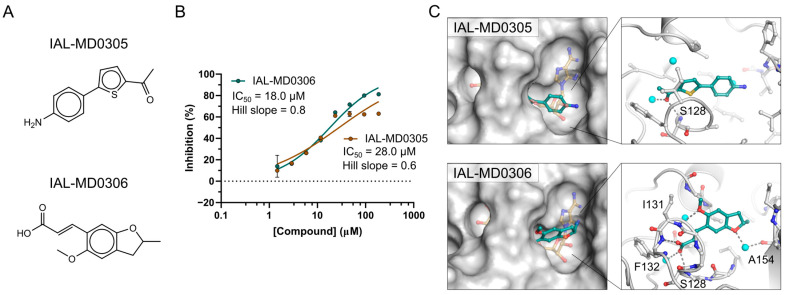
NSP3 macrodomain hit compounds identified by virtual ligand screening. (**A**) The molecular structure of the most potent hit compounds. (**B**) NSP3 Mac1 IC_50_ curves and parameters of the virtual hit compounds obtained in confirmatory HTRF assays. (**C**) Docking models of NSP3 Mac1 in complex with the hit compounds (cyan stick model). (**Right**) Surface representation showing as reference the binding mode of ADP-ribose (brown stick model in low transparency; generated by structure overlay with PDB ID 7KQP). (**Left**) Molecular interactions of the hit compounds with NSP3 Mac1. Water molecules are shown as blue spheres.

Initial ligand discovery efforts focused on the virtual screening of NSP3 Mac1. ADP-ribose was removed from the 3D structure of the bound macrodomain (PDB ID 6W02). Redocking returned the ligand bound structure with high overlap compared to the X-ray structure (RMSD 1.05 Å), confirming the docking validity (Supplementary Figure S1). A 125,000-compound virtual copy of the BioAscent library was then screened using Schrodinger Glide SP and the top 2000 compound selected for profiling. A total of 1786 compounds could be supplied by the company and were screened against NSP3 Mac1 using the HTRF assay as described below. The most potent inhibitors, IAL-MD0305 and IAL-MD0306, showed 28 µM and 18 µM IC_50_s, respectively (Figure 1A,B), although attempts at co-crystallisation and soaking did not yield experimental ligand-bound protein structures with NSP3 Mac1. Yet, the models of the macrodomain-hit compound complexes suggest a binding mode of both compounds in the open ribose-phosphate binding site of the ADP-ribose (Figure 1C). While IAL-MD0305 may be stabilised only through hydrogen bonding to the Ser128 backbone amine and hydrophobic interactions, IAL-MD0306 interacts with its carboxyl group to the backbone amines of S128, Phe132 and I131, thus rationalising its slightly higher inhibitory activity in the HTRF assay. 

### 3.2. NSP3 Macrodomain Hit Discovery by MIDAS Compound Library Screen

**Figure 2:**
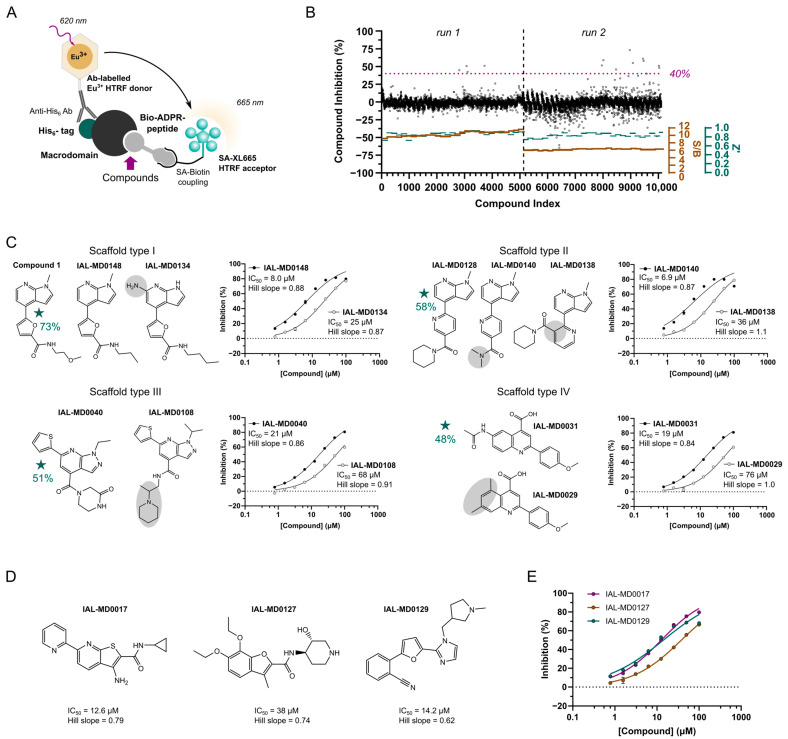
NSP3 macrodomain hit discovery by MIDAS compound library screen. (**A**) HTRF assay principle for performing the compound library screening on SARS-CoV-2 NSP3 Mac1. (**B**) Compound inhibition profile and assay quality monitoring of the MIDAS compound library screen. The cut-off for hit compounds was set to a macrodomain inhibition of ≥40%. S/B: Signal-to-Background; Z’: assay quality parameter. (**C**) Four scaffold types identified from the MIDAS screening for classifying hit compounds. Representative examples of scaffold analogues and obtained IC_50_ curves are shown. Primary screening hit compounds with respective macrodomain inhibition at 25 µM are indicated with a star. Grey circles highlight potential SAR information resulting in the observed differences on macrodomain inhibition based on IC_50_ values. All analogues with respective inhibitory activity are provided in Supplementary Table S4. (**D**) Molecular structure of the singleton compounds with their respective IC_50_ values and Hill slope parameters. (**E**) IC_50_ curves obtained for singletons in the HTRF assay.

To discover and characterise additional new hit matter for SARS-CoV-2 NSP3 Mac1, we performed in vitro primary and confirmatory screening using an established HTRF technology-based screening assay previously set-up for the characterisation of fragment hits for this target [36]. The assay involves an ADP-ribose mimic-conjugated peptide [47] that binds via its biotinylated lysine to a streptavidin-labelled XL665 HTRF acceptor fluorophore, while the macrodomain protein is complexed by its hexahistidine tag with an anti-His_6_-antibody, which itself is conjugated to the Europium HTRF donor fluorophore (Figure 2A). Binding of the macrodomain to the ADP-ribose imitating part of the peptide produces a FRET-based HTRF signal, which is disrupted by inhibitors targeting the active site of the macrodomain. ADP-ribose, which is recognised by NSP3 Mac1 with a *K*_D_ of 13 µM [48], is used as positive control, showing an IC_50_ of 1.1 µM in the HTRF assay (Supplementary Figure S2). The measured IC_50_ for ADP-ribose thus matches the ADP-ribose IC_50_ of 1.5 µM obtained in a similar set-up using the peptide in an AlphaScreen-based assay [33].

To be unbiased for potential hit matter, we screened the “Manchester Institute Diversity Set” (MIDAS), comprising 10.1 k diverse, non-covalent, and tractable small molecules. The library was screened at a compound concentration of 25 µM in two batches, with an average assay performance of 0.87 for Z’ and an S/B ratio of 10.4 in the first run and of 0.83 for Z’ and an S/B ratio of 6.2 in the second run (Figure 2B). Setting a minimum NSP3 Mac1 inhibition of 40% and a maximum assay error of 10% to be defined as a hit compound, we obtained 10 primary hit compounds, resulting in an overall hit rate of 0.1%. More specifically, one compound showed complete NSP3 Mac1 inhibition at the screening concentration, four compounds were identified with 60–70% inhibition, and the remaining five compounds showed inhibitory activity in the range from 40% to 55%. Three out of these ten hit compounds were yet excluded from further hit confirmation based on their assay interference potential. Of note is that we judged—based on our experience in medicinal chemistry and FRET-based assays, as well as references in the literature (e.g., Baell and Walters, 2014 [41])—the screening compounds for the presence of chemical features known to cause assay interference and promiscuous binding behaviour. Compounds were excluded from the hit validation processes without further biophysical testing or computational predictions when certain motifs were identified. By considering the commercial availability of the remaining hit compounds, including of respective analogues along with the primary screening results, we determined to focus on four molecular scaffolds for follow-up (Figure 2C, Supplementary Table S3) and included three compounds, IAL-MD0017, IAL-MD0127 and IAL-MD0129, as singletons in the hit confirmation process. The hit compound defining scaffold type I with a furanyl-pyrrolo[2,3-*b*]pyridine structure (Compound **1**) could not be re-supplied; however, it was followed-up with seven close analogues to obtain initial structure–activity relationships (SAR) for its binding to NSP3 Mac1. The hit compounds defining scaffold type II with a pyridinyl-pyrrolo[2,3-*b*]pyridine (IAL-MD0128), scaffold type III with a thiophenyl-pyrazolo[3,4-*b*]pyridine attached to a piperazine substituent (IAL-MD0040), and scaffold type IV with a phenylquinoline-4-carboxylic acid (IAL-MD0031), were each followed-up with dose–response titrations along with 18 (type II and IV) or 55 (type III) analogues, respectively (Supplementary Table S4). The 98 analogues were selected based on having the core of their respective scaffold type group conserved with variations of the attached ring systems, functional groups, and additions of substituents, in order to explore the amenability and plasticity of the active site of NSP3 Mac1.

### 3.3. NSP3 Macrodomain Inhibitors of Scaffold Type I 

All analogue compounds in the scaffold type I group were confirmed to inhibit NSP3 Mac1 with IC_50_ values between 4.9 µM and 25 µM (Supplementary Table S4). IAL-MD0148 that is structurally closest to the primary (non-purchasable) hit, i.e., Compound **1**, showed a macrodomain inhibition of 8.0 µM (Figure 2C top-left) and was slightly outcompeted in activity by compounds with smaller amid-containing substituents attached to the furan ring. Interestingly, IAL-MD0131 characterised by a variation of the methoxyethylamide to morpholine showed the best inhibitory activity (IC_50_ of 4.9 µM) (Supplementary Figure S3A) among this series. Furthermore, IAL-MD0134 stood out as the only compound of lower activity with an IC_50_ of 25 µM (Figure 2C top-left). Notably, its amino group being attached to the pyrrolo[2,3-*b*]pyridine core may lead to steric clashes within the active site of the macrodomain, resulting in the decrease in its inhibitory ability (Figure 2C, top-left). Of note is that all compounds in this series showed assay effects at higher compound concentrations, potentially indicating solubility-related issues, which is to be considered for compound optimisation.

### 3.4. NSP3 Macrodomain Inhibitors of Scaffold Type II 

Scaffold type II is defined by the primary screening hit IAL-MD0128, whose NSP3 Mac1 inhibition was verified in the confirmatory dose–response titrations (Supplementary Figure S3B), albeit with assay interference effects at higher compound concentrations. Its IC_50_ activity was estimated with 3.1 µM and, as such, took the lead compared to the inhibitory activities of the analogue compounds in this series with determinable IC_50_ values ranging between 6.9 µM (IAL-MD0140) and 45 µM (IAL-MD0143) (Supplementary Table S4). The attachment of the piperidinyl ethenone substituent and variations in ortho instead of in para position on the pyridine (IAL-MD0138) most notably decreased NSP3 Mac1 inhibition and, particularly, meta position substituents were not tolerated (Figure 2C top-right, Supplementary Table S4), most likely by making the compound poorly fit into the active site of the macrodomain. Moreover, any of the tested variations of the pyridinyl substituents in para position including smaller (non-)aromatic ring systems or functional group extensions did not increase the inhibitory activity of the compounds compared to the primary screening hit (Supplementary Table S4). The para substituent seems yet to be involved in macrodomain interaction, since minor variations have notable effects. While a ring opening to *N,N*-dimethylacetamide (IAL-MD0140) is well tolerated, *N*-ethyl, *N*-methylacetamide (IAL-MD0144) or a simple reduction in ring size to pyrrolidine are less favoured (IAL-MD0142). 

### 3.5. NSP3 Macrodomain Inhibitors of Scaffold Type III

Follow-up characterisation of the primary hit and 54 analogue compounds classifying to scaffold group type III provided further SAR information to target NSP3 Mac1. Only for six compounds IC_50_ values could be obtained ranging between 12.6 µM and 68 µM (Supplementary Table S4), while the primary hit (IAL-MD0040) was confirmed with an IC_50_ of 20 µM (Figure 2C bottom-left). IAL-MD0051, the best performing compound of this series, which has minor alterations of the core-attached ring systems, i.e.,thiophen (replaced with methyl-thiophen) and the piperazin-2-one (replaced with morpholine), showed slightly stronger macrodomain inhibition (IC_50_ of 12.6 µM) than the primary hit compound, yet accompanied by secondary assay effects at higher concentrations. Analogues IAL-MD0064 (IC_50_ of 25 µM) and IAL-MD0070 (IC_50_ of 16.3 µM), whose ethyl substituent is replaced with isopropyl or, additionally, the thiophen with a furan ring system, show similar activity on the macrodomain as the primary hit. In contrast, replacement of the thiophen with the slightly larger phenyl substituent (IAL-MD0123) as well as replacement of the ethyl substituent with a larger benzyl substituent (IAL-MD0124) is not tolerated, clearly showing the relevance of the size and nature of the substituents in these positions for the macrodomain binding. Moreover, linearisation of the piperazin-2-one (IAL-MD0074, IC_50_ of 25.3 µM) did not have any notable effects on macrodomain inhibitory activity. However, its replacement with an amide-linked piperidine (IAL-MD0108) was less favoured, decreasing the IC_50_ to 68 µM, while its replacement with larger substituents (which was sampled by the majority for the analogues) including dihydroquinoxalin-2-one (IAL-MD0049) was not tolerated, likely due to causing steric hindrance within the active site (Figure 2C bottom-left, Supplementary Table S4). 

### 3.6. NSP3 Macrodomain Inhibitors of Scaffold Type IV and Singletons

Scaffold type IV compounds showed overall lower inhibitory activity on the macrodomain compared to the other groups. The primary hit IAL-MD0031 was characterised with an IC_50_ of 19 µM, while derivative compounds showed either similar (IAL-MD0059 and IAL-MD0088) or notably decreased (IAL-MD0024 and IAL-MD0029) macrodomain activity (Figure 2C bottom-right, Supplementary Table S4). Interestingly, the exchange of the carboxylic acid group with carboxamide was well tolerated (IAL-MD0088, IC_50_ of 24 µM); however, its replacement with *N*-methoxy carboxamide (IAL-MD0024) was far less accepted, decreasing the IC_50_ to 68 µM, and replacement with any larger substituent (sampled by the majority of analogue compounds) resulted in a loss of inhibitory activity on the macrodomain. Furthermore, the addition of a methyl group to the quinoline core in position 7 also resulted in a decrease in macrodomain inhibitory activity (IAL-MD0029, IC_50_ of 76 µM), and even more so with a larger methoxy group (IAL-MD0030), similarly to its addition to the phenyl in position 3 (IAL-MD0094). In contrast, even bulky additions to position 2 of the quinoline core were tolerated (IAL-MD0059, IC_50_ of 22.8 µM) (Figure 2C bottom-right, Supplementary Table S4), indicating that substituents in position 2 may be directed outwards of the active site of the macrodomain. 

Finally, the dose–response titrations of the singleton hit compounds, IAL-MD0017, IAL-MD0127, and MD0129 (Figure 2D), also confirmed their inhibitory activity on NSP3 Mac1, with IC_50_s of 12.6 µM, 38.0 µM, and 14.2 µM, respectively, (Figure 2E, Supplementary Table S4) and may be considered as possible chemical matter accepted by the active site of the macrodomain for inhibitor development. 

### 3.7. Screening of FDA-Approved Compounds Reveal Antibiotics as NSP3 Macrodomain Inhibitors 

**Figure 3:**
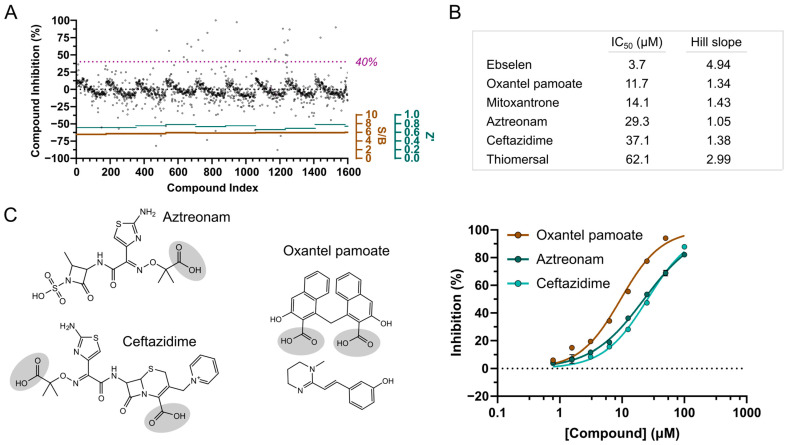
NSP3 macrodomain hit discovery by screening a library of FDA-approved compounds. (**A**) Compound inhibition profile and assay quality monitoring of the FDA-approved compound library screen. The cut-off for hit compounds was set to a macrodomain inhibition of ≥40%. S/B: signal-to-background; Z’: assay quality parameter. (**B**) NSP3 Mac1 IC_50_ values and Hill slope para-meters of best performing hit compounds in confirmatory HTRF assays. (**C**) Molecular structures and IC_50_ curves of the most promising hit compounds from the FDA-approved compound library. The carboxylic group commonly present in all three compounds is highlighted in grey circles.

In light of the benefits of discovering FDA-approved drugs as inhibitors for the target of interest, we complemented our in vitro screening approach with screening a library of 1600 FDA-approved molecules. The screening was performed with a compound assay concentration of 50 µM in one run of single shot experiments, with an average assay performance of 0.73 for Z’ and an S/B ratio of 5.8 (Figure 3A). Applying the same criteria for hit compounds as for the MIDAS screen, i.e., NSP3 Mac1 inhibition over 40% and assay error less than 10%, we obtained 30 hit compounds, which corresponds to a hit rate of 1.9% (Supplementary Table S5). Thus, compared to the MIDAS library screen, notably more compounds were identified showing assay activity, yet including both compounds of direct target inhibition and potential assay interference. Interestingly, adenine was also included among the drug molecules, yet it did not show any inhibitory activity on NSP3 Mac1, indicating that hits to be identified in the screening with the chosen parameters are likely required to undergo more interactions with the domain than ADP-ribose targeting the adenine binding site. The selection of hit compounds for follow-up was guided by structural inspection, thereby excluding compounds with features known for likely assay interference. These included biotin (NSP3 Mac1 inhibition of 107%), due to its competition with the biotinylated peptide over binding to the streptavidin-conjugated XL665 fluorophore, and in particular, compounds with large multi-ring aromatic systems such as sennoside A (112% inhibition), chlorophyllide–copper complex (106% inhibition), methacycline (87% inhibition), protoporphyrin IX (75% inhibition) and candicidin (50%). The metal-complexed compounds pyrithione zinc, cisplatin, and zinc undecylenate (68–100% inhibition) were also not prioritised for follow-up; furthermore, the high inhibition of nadide (100%) was assumed to be based on the limited stability of NAD^+^, resulting in its degradation to ADP-ribose and nicotinamide. Overall, the most active FDA-approved compounds identified from the primary screening (Supplementary Table S5) and selected for hit confirmation included the selenium and mercury-containing compounds ebselen and thiomersal (both showing 107% NSP3 Mac1 inhibition), thioctic acid (71% inhibition), avobenzone (62% inhibition), and oxantel pamoate (56% inhibition). Moreover and notably, several antibiotic compounds ranked upon the hits showing NSP3 Mac1 inhibitory activity between 108% (ceftazidime) to 43% (aztreonam). Although generally differing in structural makeup, the antibiotics were grouped into either anthracene scaffold-based compounds (methacycline and mitoxantrone) or beta-lactam-based antibiotics (ceftazidime, cephalosporin C, cefepime, ceftibuten and aztreonam).

The re-supply and testing of the 15 selected hit compounds in dose–response titration at a top assay concentration of 125 µM confirmed the activity of six drug molecules with IC_50_ values from 3.7 µM (ebselen) to 62 µM (thiomersal) (Figure 3B). The other characterised compounds did not reproduce the primary screening results, showing either inactivity over the tested concentration range or strong assay interference (carboplatin and methacycline). Ebselen showed the strongest inhibitory effect on NSP3 Mac1, yet its high Hill slope parameter and due to being generally known as a promiscuous binder indicated unspecific effects of ebselen on the target; therefore, it was not further pursued. Moreover, although an IC_50_ value of 14 µM could be estimated for mitoxantrone, strong assay interference likely based on aggregation effects could be observed. Moreover, considering the assay inactivity of the similar hit compound methacycline, mitoxantrone was also excluded from further follow-up characterisation. However, two of the three beta-lactam antibiotics, aztreonam and ceftazidime, had confirmed NSP3 Mac1 inhibition with IC_50_ values of 29 µM and 37 µM, respectively, along with oxantel pamoate with an IC_50_ value of 12 µM (Figure 3B,C). Of note is that oxantel pamoate is a two-component drug, using the embonate salt as a counterion for the oxantel base for controlling the dissolution rate of the formulation, and assuming that only one component is active on NSP3 Mac1, the IC_50_ of the latter may be around 6 µM. In all three compounds (pamoate, aztreonam, and ceftazidime), we also noted the presence of a carboxylic group (Figure 3C), potentially indicating a common motif that enables interaction with the active site of NSP3 Mac1. 

### 3.8. Aztreonam Targets the NSP3 Macrodomain Active Site Similar to MIDAS Hit Compound

**Figure 4:**
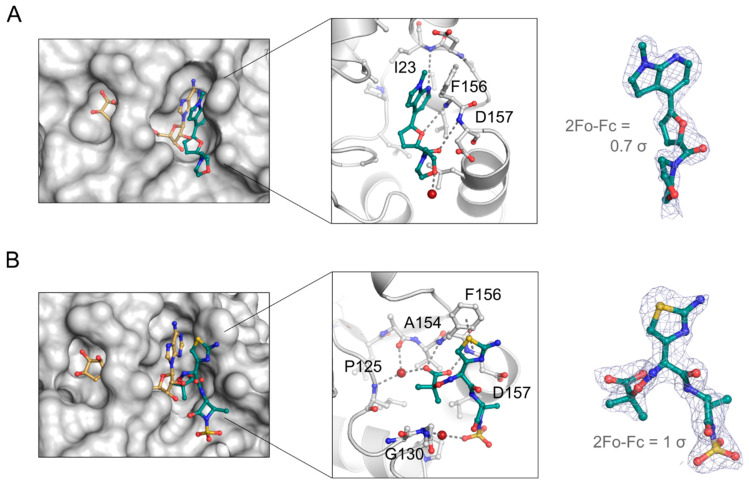
Crystallographic studies confirm the binding of the MIDAS library analogue hit IAL-MD0131 (**A**) and the FDA-approved compound aztreonam (**B**) in the active site of NSP3 Mac1. The hit compounds are shown as cyan stick model. Binding to NSP3 Mac1 in reference to ADP-ribose (brown stick model; generated by structure overlay with PDB ID 7KQP) is shown on the left, their molecular interactions with the macrodomain are shown in the middle, and the resolution of the compounds in the crystallographic map are presented on the right. Waters are shown as red spheres.

To confirm the binding of the hit compounds to SARS-CoV-2 NSP3 Mac1 and gain insights into the binding mode for structure-guided compound development, we performed co-crystallisation experiments of the macrodomain with MIDAS and FDA-approved hit compounds, whose inhibitory activity was confirmed in the dose–response titrations. For the MIDAS hit confirmation, we could determine the structure of NSP3 Mac1 in complex with IAL-MD0131 (Supplementary Table S1), which is one of the best performing MIDAS hits with an IC_50_ of 4.9 µM and belongs to the scaffold type I group. IAL-MD0131 was resolved in the crystallographic map, yet with low occupancy (Figure 4A, right). The ligand occupied the adenosine binding site of the ADP-ribose binding pocket, with its methyl-pyrrolo[2,3-*b*]pyridine moiety aligning with the adenine base of the ADP-ribose and the furan positioning in the ribose binding site (Figure 4A, left). The binding of IAL-MD0131 appears to be stabilised by hydrogen bonding of the pyrrolo[2,3-*b*]pyridine moiety to the backbone amine of Ile23 and its off-set π-π stacking to the Phe156 side chain, presenting interactions which are also established by the natural ligand ADP-ribose. Furthermore, the furan-carbonyl substructure of IAL-MD0131 enables targeting of the Phe156/Asp157 backbone amines, which is also defined as the “oxyanion” subsite of NSP3 Mac1 [36] (Figure 4A, middle). In the NSP3 Mac1:ADP-ribose-bound structure, the pro-ximal ribose interacts with this oxyanion subsite via a bridging water molecule. Chemical matter exploring this subsite with direct interaction could, therefore, presenta valuable starting point for macrodomain inhibitor development. The morpholino ring system is directed outwards of the ADP-ribose pocket and does not engage with direct interactions to the macrodomain. Functionalising this moiety for NSP3 Mac1 binding, for instance, by targeting the side-chain of Asp157, could provide possibilities to improve inhibitor potency. 

Moreover, we obtained a co-crystal structure of the macrodomain with the FDA-approved antibiotic drug aztreonam (Figure 4B, Supplementary Table S1). Aztreonam, confirming with an IC_50_ of 29 µM in dose–response titrations, was indeed identified to target the active site of the macrodomain by being well-resolved in the crystallographic map (Figure 4B, right). Compared to the ADP-ribose binding mode, aztreonam does not occupy the adenine-binding subsite and instead takes an arching confirmation into a groove adjacent below the adenosine binding pocket (Figure 4B, left). Similar to ADP-ribose and IAL-MD0131, aztreonam shows π-π stacking with Phe156, using its aminothiazol substituent. The amide beta-lactam bridges to the sulfonic acid, which is stabilised over a water-mediated contact to Gly130. Moreover, aztreonam hydrogen bonds to the oxyanion subsite of NSP3 Mac1 with its carboxylic acid that additionally coordinates over a water molecule to the backbone amines of Ala154 and Pro125 (Figure 4B, middle). The hydrogen bond interactions established by the carboxylic acid within the proximal ribose binding pocket may contribute to the binding affinity and, as a result, to the observed inhibitory activity of aztreonam. The carboxylic acid group was a common functional group among the discovered FDA-approved hit compounds which confirmed in dose–response titrations with confidence; therefore, it is tempting to assume that, also in these compounds, the carboxylic acid group contributes to NSP3 Mac1 inhibition via interaction with the oxyanion subsite. 

## 4. Discussion

SARS-CoV-2 NSP3 Mac1 presents an alternative, promising target for the development of a new type of antiviral agent [26]. While characterised antiviral drugs acting against host-derived targets still need to prove their effectiveness in the clinic, several compounds targeting proteins involved in the viral life cycle and/or pathogenesis are approved for treatment, with the RNA-dependent RNA polymerase and viral protease inhibitors being most widely used at present [10,11]. However, recent studies demonstrated that resistances to remdesivir and nirmatrelvir (marketed as Paxlovid™) can arise via multiple pathways [49,50], rendering treatments ineffective and requiring the development of alternative strategies. We supported these efforts by performing a computational screen along with an in vitro compound library screen of experimental small molecules and FDA-approved drugs against NSP3 Mac1. 

Virtual screening of a medium-sized library (125,000) of small molecules from BioAscent yielded two novel inhibitors with micromolar potency, IAL-MD0305 and IAL-MD0306. Efforts to improve the compound potency using structure-based drug design was hampered by the failure of either compound to yield ligand-bound crystal structures, and, as the hits from the in vitro screening of the MIDAS library were more attractive, effort shifted to those hits. 

Complementation of this computational approach with the in vitro screening of ~12,000 compounds allowed us to gain more insights into the chemical matter amenable for the active site of NSP3 Mac1. The established HTRF assay [36] allowed a reliable identification of hit compounds, considering that we observed a high reproducibility of primary screening hits confirming in dose–response titrations of the re-supplied compounds. Taken together, the in vitro screening approach was performed with an overall hit rate of 0.3%, yielding diverse hits with inhibitory activity of both, experimentally screening molecules to explore the chemical space and developed FDA-approved drugs. Four MIDAS library-based compounds defined by different scaffold types were followed-up by the characterisation of close analogues. Despite limitations in compound availability that would allow a step-by-step analysis of the contributions of individual functional groups and ring-systems attached to the core scaffold motif to the inhibitory activity on NSP3 Mac1, the characterisation of the selected analogues provided valuable SAR information, and the tested substituents extending and/or modifying the core scaffolds allowed preliminary exploration of the active site for NSP3 Mac1 targeting. Consistent observations for related analogues regarding effects on the inhibitory activity of similar scaffold modifications also increased our confidence for the discovered hit compounds as being true NSP3 Mac1 inhibitors. Of all characterised analogues, compounds classified to the scaffold type I group generally showed the strongest NSP3 Mac1 inhibition, with IAL-MD0131 also being crystallographically confirmed for target binding. The IAL-MD0131 co-crystal structure may also allow us to infer the binding mode of the other characterised analogues within this group, considering their close structural similarity. As such, based on the orientation of the pyrrolo[2,3-*b*]pyridine in the crystal structure, attachment of the amino group in compound IAL-MD0134 leads as assumed to a sterical clash within the adenosine binding pocket, rationalising the observed drop of the IC_50_ compared to the primary hit. The scaffold type I core motif, i.e., the pyrrolo[2,3-*b*]pyridine, is moreover present in variations in scaffold types II and III, which eases molecular docking studies for defining the binding site and orientation of the molecules for the structure-guided design of follow-up compounds. Further structural and biophysical studies that generally confirm the binding along with providing NSP3 Mac1 targeting information of compounds belonging to the scaffold type II–IV groups would greatly foster and complement the in vitro-based analogue characterisation.

Re-purposing drugs for new targets has the potential to considerably accelerate the drug discovery and development process. Screening a library of FDA-approved molecules led to the discovery of several new hits which have not been described in the literature so far. This may partly be due to the different composition of the available library and partly due to the different screening assay format, which hampers comparability. Cefatrizine, dasatinib, and dihydralazine were previously described as NSP3 Mac1 inhibitors [30,51], yet this could not be confirmed since they were not included in our library. Interestingly, hydralazine was among the screened drug molecules that showed no NSP3 Mac1 inhibition at the set screening concentration, thus potentially indicating that the second hydrazine moiety notably contributes to the inhibitory activity. However, cefaclor, rabeprazole, and telmisartan were also included in our FDA-approved compound library, for which we could yet not measure any inhibitory activity on NSP3 Mac1 in our set-up compared to other studies [51]. Furthermore, oxaprozin may present an example that could not be identified in our screen for NSP3 Mac1 targeting, which is likely due to the assay format. The binding of oxaprozin to NSP3 Mac1 was discovered by protein-based nuclear magnetic resonance (NMR) screening experiments [37]. However, with the HTRF assay employed in this study particularly detecting compounds displacing the ADP-ribose-conjugated peptide from the active site of the macrodomain, the assay is less sensitive for identifying allosteric binders that do not induce notable conformational changes of the active site. However, we could confirm the inhibitory activity of suramin, which was identified as NSP3 Mac1 hit, also by using a FRET-based screening assay [52]; however, we did not follow suramin up due to its complex molecular structure and the potential overlap of these two studies. Furthermore, thiomersal inhibited NSP3 Mac1 with an IC_50_ of 62.1 µM, which was also identified as an inhibitor for MacroD1 with an IC_50_ of 5.2 µM in an AlphaScreen-based assay format [53]. Possibly, thiomersal is able to target both macrodomains due to the structural conservation of their folds.

Compared to the previous studies, our screen identified a series of antibiotic drug molecules as inhibitors for NSP3 Mac1. Although not all of them showed reproducibility with confidence in the confirmatory dose–response titrations, we verified ceftazidime and aztreonam with micromolar inhibitory activity on NSP3 Mac1. Moreover, aztreonam was confirmed for true NSP3 Mac1 targeting by crystallographic studies, providing insights into its binding mode, which would have been potentially challenging to predict by computational docking studies due to exploring regions of the macrodomain outside of the well-defined ADP-ribose binding pocket. Aztreonam uses for NSP3 Mac1 inhibition motifs, including Phe156 targeting with an aromatic system and the interaction with the oxyanion site using a carboxylate group, as has been previously observed by low-molecular fragments for targeting the domain [36]. Of note is that, albeit being a conserved fold, NSP3 Mac1 is subjected to evolutionary amino acid substitutions that have effects on ADP-ribose binding and enhance the ability of SARS-CoV-2 to counteract host immune response [54]. It is therefore conceivable that substitutions, e.g., of Phe156 or in the oxyanion site, can evolve, which may impair inhibitor binding as drug resistance strategy of the virus. Also of note is that Phe156 (also targeted by the MIDAS hit compound IAL-MD0131) is unique to SARS-CoV-2 amongst the β-coronaviruses; however, it is found present in human macrodomains including MacroD2 and PARP14 MD3. While being potentially advantageous for the development of SARS-CoV-2 NSP3 Mac1 inhibitors that are selective over other viral Mac1 variants, additional inhibitor interactions need to be considered and elaborated to achieve selectivity over human macrodomains. Compared to fragments, aztreonam also provides an underlying scaffold linking these motifs that additionally extends beyond the ADP-ribose binding site, which could potentially be exploited for selectivity purposes over other macrodomains [26]. As an FDA-approved drug, aztreonam furthermore possesses good pharmacokinetic properties, and merging it with the fragment chemical matter could be considered to develop potent NSP3 Mac1 inhibitors in future studies.

## Data Availability

Crystallography atomic coordinates and structure factors are deposited in the PDB (https://www.rcsb.org, accessed on 11 January 2023) under the following accession codes: 8C19 and 8C1A. All data supporting the findings of this study are available within the Article or the Supplementary Information. Any further information will be provided upon request to the corresponding authors.

## Supplementary Materials

The following supporting information can be downloaded at: https://www.mdpi.com/article/10.3390/pathogens12020324/s1, Figure S1: Redocking of ADP-ribose into SARS-CoV-2 NSP3 Mac1; Figure S2: ADP-ribose is used as positive control and reference compound in the HTRF-based assay for inhibitor screening and characterisation for SARS-CoV-2 NSP3 Mac1.; Figure S3: Dose-response titrations of MIDAS compound library hits leading regarding NSP3 Mac1 inhibitory activity.; Table S1: Data collection and refinement statistics for crystal structures described in this study.; Table S2: Top hit compounds from virtual screening approach.; Table S3: Hit compounds from MIDAS compound library screen.; Table S4: Confirmation of hit compounds and analogues for SAR information.; Table S5: Hit compounds from FDA-approved compound library screen. Excel file: Supplementary Tables S2–4 with molecular structures.
