# Supplementary material for: Discovery and Development Strategies for SARS-CoV-2 NSP3 Macrodomain Inhibitors
Source: Pathogens. 2023 Feb 15;12(2):324. doi: 10.3390/pathogens12020324 (PMC9965906; doi:10.3390/pathogens12020324)
Supplement: Supplementary file 1 [file pathogens-12-00324-s001.zip › SupplementaryInformation_Schuller et al_revised.pdf]

Discovery and development strategies for SARS-CoV-2 NSP3 macrodomain inhibitors

Marion Schuller[1*], Tryfon Zarganes-Tzitzikas[2], James Bennett[2], Stephane De Cesco[2], Daren Fearon[3,4], Frank von Delft[2,3,4,5,6], Oleg Fedorov[2], Paul E. Brennan[2], Ivan Ahel[1*]

[1]*Sir William Dunn School of Pathology, University of Oxford, Oxford OX1 3RE, UK*

[2]*Centre for Medicines Discovery, University of Oxford, Headington OX3 7DQ, UK*

[3]*Diamond Light Source Ltd., Harwell Science and Innovation Campus, Didcot OX11 0DE, UK*

[4]*Research Complex at Harwell, Harwell Science and Innovation Campus,Didcot OX11 0FA, UK*

[5]*Structural Genomics Consortium, University of Oxford, Headington OX3 7DQ, UK*

[6]*Department of Biochemistry, University of Johannesburg, Auckland Park 2006, South Africa*

*Corresponding author: ivan.ahel@path.ox.ac.uk; marion.schuller@path.ox.ac.uk

SUPPLEMENTARY FIGURES AND TABLES

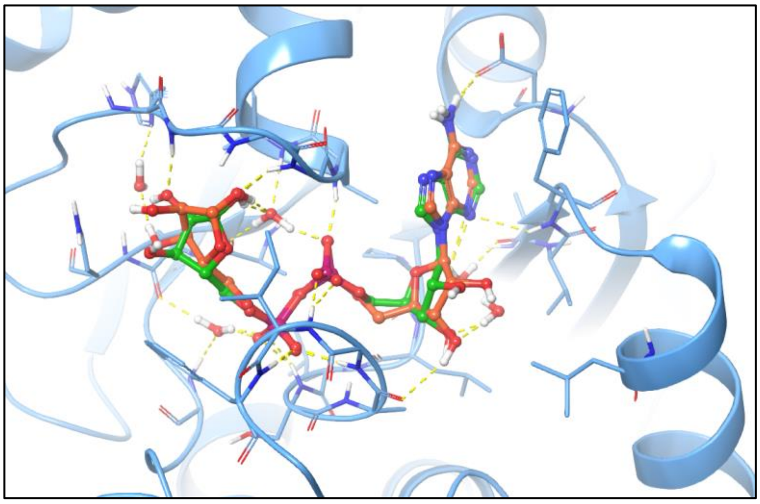

Figure S1. Redocking of ADP-ribose (orange sticks) in the NSP3 macrodomain (blue ribbon and sticks) yields a ligand structure with an overlap of RMSD 1.05 Å compared to the X-ray structure (green sticks).

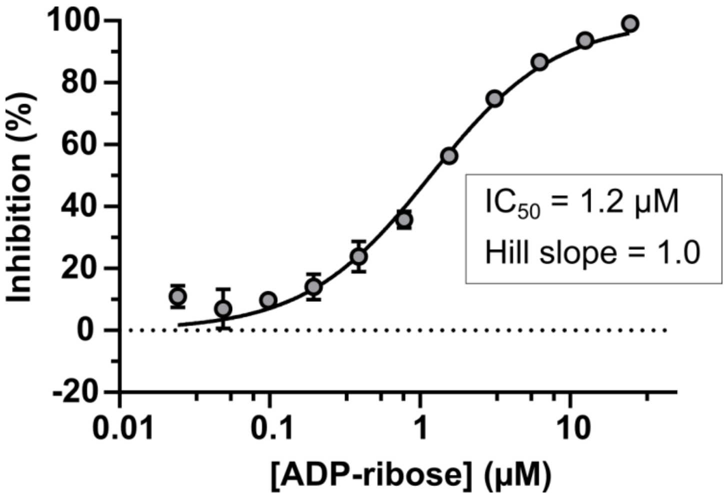

Figure S2. ADP-ribose is used as positive control and reference compound in the HTRF-based assay for inhibitor screening and characterisation for SARS-CoV-2 NSP3 Mac1. A dose-response titration is shown with IC_{50} and Hill slope parameter provided in the inset.

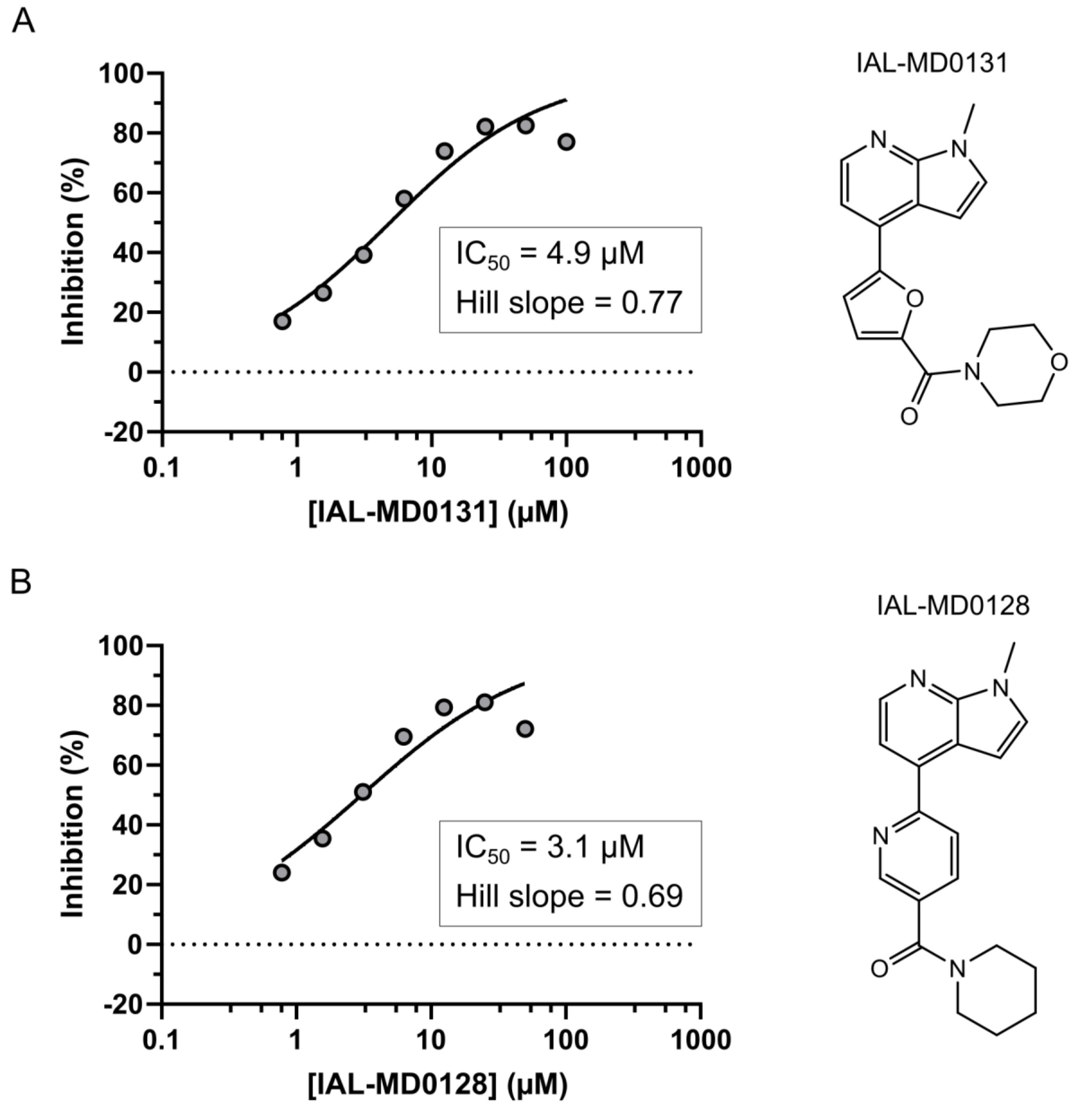

Figure S3. Dose-response titrations of MIDAS compound library hits leading regarding NSP3 Mac1 inhibitory activity. (A) IAL-MD0131 belongs to the scaffold type I group. **(B)** IAL-MD0128 belongs to scaffold type II group. Data point for highest concentration of IAL-MD0128 was excluded. Respective IC_{50} and Hill slope parameters are provided in the insets, the molecular structures on the right.

Supplementary Table S1. Data collection and refinement statistics for crystal structures described in this study.

	SARS-CoV-2 NSP3 Mac1 in complex with IAL-MD0131	SARS-CoV-2 NSP3 Mac1 in complex with aztreonam
**PDB accession code**	**8C19**	**8C1A**
**Data Collection**		
Synchrotron/beam line	DLS/I03	DLS/I03
Wavelength (Å)	0.896844	0.97623
Space group	$P\ 4_3$	$P\ 4_3$
a (Å)	88.604	88.4745
b (Å)	88.6043	88.4745
c (Å)	39.8265	39.139
α (°)	90	90
β (°)	90	90
γ (°)	90	90
Content of AU	2	2
Resolution (Å) [a]	39.63 - 1.95 (2.02 - 1.95)	62.56 - 1.9 (1.94 - 1.9)
R_{sym} (%) [a,b]	0.318 (2.285)	0.024 (0.044)
I/σ(I)	5.72 (1.19)	64.34 (21.01)
Completeness (%) [a]	100.0 (100.0)	95.2 (72.1)
Redundancy [a]	13.4 (13.9)	10.8 (4.8)
$CC_{1/2}$ (%) [a]	0.996 (0.603)	1 (0.983)
Unique reflections [a]	22915 (1607)	22970 (1085)
**Refinement**		
R_{cryst} (%) [c]	0.1904 (0.3096)	0.1806 (0.2010)
R_{free} (%) [d]	0.2313 (0.3237)	0.2238 (0.2526)
RMSD bond length (Å)	0.010	0.009
RMSD bond angle (°)	1.61	1.68
Amino acids [e]	334 (28.66)	334 (20.47)
Water [e]	133 (32.13)	138 (23.40)
Ligands [e]	1 (49.66)	1 (32.25)
Ions [e]	0	0
**Ramachandran plot**		
Favoured (%)	98.18	98.78
Allowed (%)	1.82	0.91
Disallowed (%)	0.00	0.30

(a) Data for the highest resolution shell are given in parentheses.
(b) R_{sym} = Σ|/-</>|/Σ/, where / is measured density for reflections with indices *hkl*.
(c) R_{cryst} = Σ||Fobs| - |Fcalc||/Σ|Fobs|.
(d) R_{free} has the same formula as R_{cryst}, except that calculation was made with the structure factors from the test set.
(e) Number of atoms followed the average B factor in brackets.

Supplementary Table S2. Top hit compounds from virtual screening approach. Only hit compounds showing more than 30% NSP3 macrodomain inhibition are listed.

Compound library ID	Compound internal ID	Nsp3 macrodomain inhibition at 25 µM (%)	Error (%)	IC_{50} in µM (Inhibition at top screening concentration of 187 µM)	SMILES
BCC0086077	---	67.8	0.7	---	Cc1cc2nn(-c3ccccc3)nc2cc1N
BCC0122573	IAL-MD0306	63.7	0.1	18.0 (81.3%)	COc1cc2c(cc1/C=C/C(=O)O)OC(C)C2
BCC0111915	---	62.9	10.9	---	O=c1c(O)c(-c2cccs2)oc2ccccc12
BCC0119417	---	59.3	1.3	---	CC(=O)c1ccc(-c2ccc(N)cc2)o1
BCC0125028	---	57.5	0.9	---	O=c1ccsn1-c1ccc(F)c(Cl)c1
BCC0121661	---	56.9	0.1	---	Nc1ccc2c(c1)C/C(=C/c1ccco1)C2=O
BCC0091906	---	56.2	0.2	---	Nc1ccc2nc3c(=O)[nH]c(=O)[nH]c3nc2c1
BCC0115778	IAL-MD0305	56.2	3.5	28.0 (63.1%)	CC(=O)c1ccc(-c2ccc(N)cc2)s1
BCC0002149	---	47.2	0.4	---	COc1cc(/C=C2\Oc3cc(O)ccc3C2=O)ccc1O
BCC0083752	---	46.8	2.9	---	CN(C)c1ccc(-c2ccc3ccccc3n2)cc1
BCC0117785	---	39.3	0.4	---	COc1cc(/C=C/C(=O)c2cc3ccccc3o2)cc(Br)c1O
BCC0093974	---	38.6	1.3	---	Oc1cc2ccccc2nc1-c1ccccc1
BCC0032304	---	36.4	1.6	---	CCN1C(=O)c2cccc3c(S(=O)(=O)O)ccc1c23
BCC0071692	---	35.0	2.0	---	Oc1nc2ccccc2nc1-c1c[nH]c2ccccc12
BCC0019152	---	32.7	0.6	---	CN(C)S(=O)(=O)c1ccc2nc(C(=O)O)n(O)c2c1
BCC0030992	---	31.6	0.8	---	O=c1cc(-c2ccc(Cl)cc2)oc2cc(OCC(O)CS(=O)(=O)c3ccc(Cl)cc3)ccc12
BCC0049478	---	30.6	0.6	---	Cc1cccn2c(=O)c3cc(C(=O)O)n(C)c3nc12
BCC0067629	---	30.5	0.3	---	CN1C(=O)c2ccc(N3CCCC3)cc2C1=O
BCC0105824	---	30.1	1.4	---	Nc1ccc2oc(-c3cccnc3)nc2c1

Table S3. Hit compounds from MIDAS compound library screen.

Compound library ID	Compound internal ID	NSP3 Mac1 inhibition at 25 µM (%)	Error (%)	SMILES	Scaffold type
ARUK4000671-001-001	---	106.5	0.18	O=C(Nc1ccc2cc[nH]c2c1)c1csc(n1)NCC1CCCO1	Excluded based on assay interference potential
ARUK4007189-001-001	---	73.1	0.33	COCCNC(=O)c1ccc(o1)-c1ccnc2n(ccc12)C	I
ARUK4004310-001-001	---	61.0	7.02	CC(=O)N1CCc2nc(c(c(c2C1)-c1cccc(c1)-c1ccco1)C#N)N	Excluded based on assay interference potential
ARUK4010026-001-001	IAL-MD0017	58.7	0.34	Nc1c(sc2nc(ccc12)-c1ccccn1)C(=O)NC1CC1	Other
ARUK4007069-001-001	IAL-MD0128	58.0	0.82	Cn1ccc2c(ccnc12)-c1ccc(cn1)C(=O)N1CCCCC1	II
ARUK4001517-001-001	IAL-MD0129	55.0	0.53	CN1CCC(C1)Cn1ccnc1-c1ccc(o1)-c1ccccc1C#N	Other
ARUK4002661-001-001	IAL-MD0040	51.0	0.71	CCn1ncc2c(cc(nc12)-c1cccs1)C(=O)N1CCNC(=O)C1	III
ARUK4002695-001-001	IAL-MD0031	48.0	0.53	COc1ccc(cc1)-c1cc(c2cc(ccc2n1)NC(=O)C)C(=O)O	IV
ARUK4000702-001-001	---	45.8	0.92	NC(=O)[C@@H]1CCCN1C(=O)c1cc(ccc1Cl)-n1cccc1	Excluded based on assay interference potential
ARUK4007403-001-001	IAL-MD0127	40.0	2.15	CCOc1ccc2c(c(oc2c1OCC)C(=O)N[C@@H]1CCNC[C@H]1O)C	Other

Table S4. Confirmation of hit compounds and analogues for SAR information.

Internal compound ID	IC_{50} in µM	Inhibition at top concentration of 125 µM (%)	SMILES
**Scaffold type I**			
IAL-MD0131	4.9	Not measurable due to compound precipitation	O=C(C1=CC=C(C2=C3C(N(C)C=C3)=NC=C2)O1)N4CCOCC4
IAL-MD0147	6.8	Not measurable due to compound precipitation	O=C(NCC)C1=CC=C(C2=C3C(N(C)C=C3)=NC=C2)O1
IAL-MD0132	7.2	Not measurable due to compound precipitation	O=C(NC1CC1)C2=CC=C(C3=C4C(N(C)C=C4)=NC=C3)O2
IAL-MD0130	7.6	Not measurable due to compound precipitation	O=C(NC(C)C)C1=CC=C(C2=C3C(N(C)C=C3)=NC=C2)O1
IAL-MD0148	7.7	Not measurable due to compound precipitation	O=C(NCCC)C1=CC=C(C2=C3C(N(C)C=C3)=NC=C2)O1
IAL-MD0133	8.5	Not measurable due to compound precipitation	O=C(N(C)C)C1=CC=C(C2=C3C(N(C)C=C3)=NC=C2)O1
IAL-MD0134	25.3	77.8	O=C(NCCCC)C1=CC=C(C2=C3C(NC=C3)=NC(N)=C2)O1
**Scaffold type II**			
IAL-MD0128	3.1	Not measurable due to compound precipitation	O=C(C1=CN=C(C2=C3C(N(C)C=C3)=NC=C2)C=C1)N4CCCCC4
IAL-MD0140	6.9	Not measurable due to compound precipitation	O=C(N(C)C)C1=CN=C(C2=C3C(N(C)C=C3)=NC=C2)C=C1
IAL-MD0145	14.0	81.1	CN1C=CC2=C(C3=NC=C(CN4C=CN=C4)C=C3)C=CN=C21
IAL-MD0141	25.2	81.3	O=C(N1CC(N(C)C)CC1)C2=CC=C(C3=C4C(NC=C4)=NC=N3)C=C2.O=CO
IAL-MD0138	36.0	79.4	O=C(C1=CC=CN=C1C2=C3C(N(C)C=C3)=NC=C2)N4CCCCC4
IAL-MD0143	45.0	71.9	O=C(C1=CC=C(C2=C3C(NC=C3)=NC=N2)C=C1)N4CCN(C(C)=O)CC4
IAL-MD0136	Not measurable	49.7	O=C(N)C1=C(C2=C3C(N(C)C=C3)=NC=C2)N=CC=C1
IAL-MD0142	Not measurable	37.9	O=C(C1=CN=C(C2=C3C(N(C)C=C3)=NC=C2)C=C1)N4CCCC4
IAL-MD0137	Not measurable	25.8	O=C(C1=CN=CC(C2=C3C(N(C)C=C3)=NC=C2)=N1)N4CCCCC4
IAL-MD0144	Not measurable	23.8	O=C(N(CC)C)C1=CN=C(C2=C3C(N(C)C=C3)=NC=C2)C=C1
IAL-MD0139	Not measurable	21.7	O=C(C1=CC(C2=C3C(N(C)C=C3)=NC=C2)=NC=C1)N4CCCC4
IAL-MD0135	Not measurable	18.3	O=C(C1=NC(C2=C3C(N(C)C=C3)=NC=C2)=CN=C1)N(C)C
IAL-MD0114	Not measurable	13.4	CC1(CCC(=O)NC1)C(=O)N2CCCC(C2)C3=CNC=4N=CC=CC34
IAL-MD0084	Not measurable	13.2	COC=1N=CC=CC1C(=O)N2CCC(CC2)C3=CNC=4N=CC=CC34
IAL-MD0078	Not measurable	10.5	CC(=O)NC=1C=CC=C(C1)C(=O)N2CCC(CC2)C3=CNC=4N=CC=CC34

IAL-MD0115	Not measurable	9.3	CNC(=O)C=1C=CC(=CN1)C(=O)N2CCC(CC2)C3=CNC=4N=CC=CC34
IAL-MD0110	Not measurable	7.8	O=C(N1CCC(CC1)C2=CNC=3N=CC=CC23)C=4C=CC(NCC5CC5)=NC4
IAL-MD0146	Not measurable	3.7	O=C(C1=CN=CC(C2=C3C(N(C)C=C3)=NC=C2)=N1)N4CCN(C)CC4
**Scaffold type III**			
IAL-MD0051	12.6	59.8	CC(C)N1N=CC=2C(=CC(=NC12)C3=CC=C(C)S3)C(=O)N4CCOCC4
IAL-MD0070	16.3	83.2	CC(C)N1N=CC=2C(=CC(=NC12)C3=CC=CO3)C(=O)N4CCNC(=O)C4
IAL-MD0040	20.6	81.0	CCN1N=CC=2C(=CC(=NC12)C3=CC=CS3)C(=O)N4CCNC(=O)C4
IAL-MD0064	25.2	78.7	CC(C)N1N=CC=2C(=CC(=NC12)C3=CC=CS3)C(=O)N4CCNC(=O)C4
IAL-MD0074	25.3	77.7	CCN1N=CC=2C(=CC(=NC12)C3=CC=CS3)C(=O)NCC(=O)N(C)C
IAL-MD0108	67.8	60.5	CC(C)N1N=CC=2C(=CC(=NC12)C3=CC=CS3)C(=O)NCC(C)N4CCCCC4
IAL-MD0116	Not measurable	55.8	COCCN(CC(=O)N)C(=O)C=1C=C(N=C2N(C)N=C(C)C12)C3=CC=CS3
IAL-MD0081	Not measurable	53.0	CNC(=O)CN(C)C(=O)C=1C=C(N=C2N(C)N=C(C)C12)C3=CC=CS3
IAL-MD0038	Not measurable	50.5	CCN1N=CC=2C(=CC(=NC12)C3=CC=CS3)C(=O)NC=4C=C(Cl)C=CC4OC
IAL-MD0086	Not measurable	47.7	CC(C)N1N=CC=2C(=CC(=NC12)C3=CC=CS3)C(=O)N4CCCC(C)(O)C4
IAL-MD0045	Not measurable	47.5	CC1=NN(C)C=2N=C(C=C(C(=O)N3CCN(CC3)C(=O)C4CC4)C12)C=5C=CC=CC5
IAL-MD0083	Not measurable	47.4	CC(C)N1N=CC=2C(=CC(=NC12)C3=CC=CS3)C(=O)N4CCCC4
IAL-MD0073	Not measurable	47.0	CC(C)N1N=CC=2C(=CC(=NC12)C3=CC=CS3)C(=O)N(C)CC=4C=C(C)ON4
IAL-MD0080	Not measurable	45.9	CC(C)N1N=CC=2C(=CC(=NC12)C3=CC=CS3)C(=O)N4CCCCC4C(=O)N5CCCC5
IAL-MD0062	Not measurable	42.2	CCN1N=CC=2C(=CC(=NC12)C3=CC=CS3)C(=O)N(C)CC(=O)NCC4=CC=CO4
IAL-MD0054	Not measurable	41.5	CC(=O)N1CCN(CC1)C(=O)C=2C=C(N=C3N(C)N=C(C)C23)C4=CC=CS4
IAL-MD0107	Not measurable	40.5	CC(C)N1N=CC=2C(=CC(=NC12)C3=CC=CS3)C(=O)N4CCC(C)(O)CC4
IAL-MD0071	Not measurable	36.8	CCN1N=CC=2C(=CC(=NC12)C3=CC=CS3)C(=O)N(CC=4C=CC=CN4)C(C)C
IAL-MD0106	Not measurable	32.1	CC(C)N1N=CC=2C(=CC(=NC12)C3=CC=CS3)C(=O)N4CCC(C)(CO)CC4
IAL-MD0075	Not measurable	30.9	CC(C)N1N=CC=2C(=CC(=NC12)C3=CC=CS3)C(=O)N4CCNC(=O)C4(C)C
IAL-MD0044	Not measurable	30.2	CC(C)N1N=CC=2C(=CC(=NC12)C=3C=CC=4OCCOC4C3)C(=O)N5CCNC(=O)C5
IAL-MD0087	Not measurable	22.3	CC(C)N1N=CC=2C(=CC(=NC12)C3=CC=CS3)C(=O)N4CC(C)OCC4(C)C
IAL-MD0042	Not measurable	20.9	CCN1N=CC=2C(=CC(=NC12)C3=CC=CS3)C(=O)N(C)CC=4C=CC=CC4C
IAL-MD0032	Not measurable	20.3	CC(C)N1N=CC=2C(=CC(=NC12)C3=CC=CS3)C(=O)NC=4C=CC=C(NC(=O)C)C4

IAL-MD0095	Not measurable	19.8	CC(C)N1N=CC=2C(=CC(=NC12)C3=CC=CS3)C(=O)NC(C)C#N
IAL-MD0055	Not measurable	19.4	CC(C)N1N=CC=2C(=CC(=NC12)C3=CC=CS3)C(=O)NC=4C=CN=CC4
IAL-MD0104	Not measurable	18.4	CC(C)N1N=CC=2C(=CC(=NC12)C3=CC=CS3)C(=O)N(C)CC=4C=CC=C(O)C4
IAL-MD0121	Not measurable	17.8	CC1=NN(C)C=2N=C(C=C(C(=O)NC3CCOCC3)C12)C4=CC=CS4
IAL-MD0092	Not measurable	17.1	CCN1N=CC=2C(=CC(=NC12)C3=CC=CS3)C(=O)NCCNC(=O)C
IAL-MD0048	Not measurable	15.8	CC1=NN(C)C=2N=C(C=C(C(=O)NCC=3C=CC=CN3)C12)C4=CC=CS4
IAL-MD0049	Not measurable	14.3	CC(C)N1N=CC=2C(=CC(=NC12)C3=CC=CS3)C(=O)N4CC(=O)NC=5C=CC=CC45
IAL-MD0066	Not measurable	13.5	CCN1N=CC=2C(=CC(=NC12)C3=CC=CS3)C(=O)N4CCCC4C=5C=CC(OC)=CC5
IAL-MD0033	Not measurable	12.1	COC=1C=CC=C(NC(=O)C=2C=C(N=C3N(N=CC23)C(C)C)C4=CC=CS4)C1
IAL-MD0026	Not measurable	11.1	CCN1N=CC=2C(=CC(=NC12)C3=CC=CS3)C(=O)NC4=NC=C(C)S4
IAL-MD0090	Not measurable	11.0	CC(C)N1N=CC=2C(=CC(=NC12)C3=CC=CS3)C(=O)NC=4C=CN(C)N4
IAL-MD0072	Not measurable	9.8	CC(C)N1N=CC=2C(=CC(=NC12)C3=CC=CS3)C(=O)N4CCN5C=CC=C5C4C
IAL-MD0117	Not measurable	9.7	CCN1N=CC=2C(=CC(=NC12)C3=CC=CS3)C(=O)NCC=4C=CC(OC)=NC4
IAL-MD0065	Not measurable	9.5	CC(C)N1N=CC=2C(=CC(=NC12)C3=CC=CS3)C(=O)N(C)CC=4C=CC=5OCOC5C4
IAL-MD0037	Not measurable	9.3	COC=1C=CC(NC(=O)C=2C=C(N=C3ON=C(C)C23)C=4C=CC(C)=CC4)=CC1
IAL-MD0120	Not measurable	8.9	CCN1N=CC=2C(=CC(=NC12)C3=CC=CS3)C(=O)NC(C)C=4C=CN=CC4
IAL-MD0093	Not measurable	7.7	CCCN(CC(=O)NC=1C=CC=CC1C)C(=O)C=2C=C(N=C3N(CC)N=CC23)C4=CC=CS4
IAL-MD0097	Not measurable	7.7	CCN1N=CC=2C(=CC(=NC12)C3=CC=CS3)C(=O)NC=4C=CC(=CC4)C(=O)N
IAL-MD0056	Not measurable	7.0	CCN1N=CC=2C(=CC(=NC12)C3=CC=CS3)C(=O)N4CCC(CC4)C(O)C=5C=CC=CC5
IAL-MD0061	Not measurable	7.0	CCN1N=CC=2C(=CC(=NC12)C3=CC=CS3)C(=O)NC=4C=CC=C(C4)C5=NN=CO5
IAL-MD0119	Not measurable	6.3	CCN1N=CC=2C(=CC(=NC12)C3=CC=CS3)C(=O)NC(C)C=4C=CC=CN4
IAL-MD0096	Not measurable	3.9	CCN1N=CC=2C(=CC(=NC12)C=3C=CC=CC3)C(=O)N4CCCC(C)C4
IAL-MD0060	Not measurable	1.9	CC(C)N1N=CC=2C(=CC(=NC12)C3=CC=CS3)C(=O)N4CCC=5C=CC=CC5C4
IAL-MD0123	Not measurable	1.8	CC(C)N1N=CC=2C(=CC(=NC12)C=3C=CC=CC3)C(=O)N4CCNC(=O)C4
IAL-MD0018	Not measurable	Not measurable	CC(C)N1N=CC=2C(=CC(=NC12)C=3C=CC=CC3)C(=O)NCC4=CC=CS4
IAL-MD0025	Not measurable	Not measurable	CCN1N=CC=2C(=CC(=NC12)C=3C=CC=CC3)C(=O)NC=4C=CC=C(NC(=O)C)C4
IAL-MD0050	Not measurable	Not measurable	CCN1N=CC=2C(=CC(=NC12)C=3C=CC=CC3)C(=O)N4CCOCC4
IAL-MD0069	Not measurable	Not measurable	CCN1N=CC=2C(=CC(=NC12)C=3C=CC=CC3)C(=O)N4CCN(CC4)C(=O)C=5C=CC=CC5
IAL-MD0082	Not measurable	Not measurable	CC(C)N1N=CC=2C(=CC(=NC12)C=3C=CC=CC3)C(=O)N4CCN(CC4)C(=O)C

IAL-MD0109	Not measurable	Not measurable	CCN1N=CC=2C(=CC(=NC12)C=3C=CC=CC3)C(=O)N(CC=4C=CC=CN4)C(C)C
IAL-MD0124	Not measurable	Not measurable	CC=1C=CC(CN2N=CC=3C(=CC(C)=NC23)C(=O)N4CCNC(=O)C4)=CC1
**Scaffold type IV**			
IAL-MD0031	19.3	81.3	COC=1C=CC(=CC1)C=2C=C(C(=O)O)C=3C=C(NC(=O)C)C=CC3N2
IAL-MD0059	22.8	63.0	CC(=O)NC=1C=CC(C=CC=2C=C(C(=O)O)C=3C=C(C)C=CC3N2)=CC1
IAL-MD0088	24.0	79.4	COC=1C=CC(=CC1)C=2C=C(C(=O)N(C)C)C=3C=CC=CC3N2
IAL-MD0024	68.0	60.3	CONC(=O)C=1C=C(N=C2C=CC=CC12)C=3C=CC(OC)=CC3
IAL-MD0029	75.9	61.2	COC=1C=CC(=CC1)C=2C=C(C(=O)O)C=3C(C)=CC(C)=CC3N2
IAL-MD0030	Not measurable	44.7	COC=1C=CC(=CC1)C=2C=C(C(=O)O)C=3C=CC(OC)=CC3N2
IAL-MD0103	Not measurable	38.4	COC=1C=CC(=CC1)C=2C=C(C(=O)NC3CC3)C=4C=CC=CC4N2
IAL-MD0094	Not measurable	31.9	COC=1C=CC(=CC1OC)C=2C=C(C(=O)O)C=3C=CC=CC3N2
IAL-MD0118	Not measurable	25.6	COC=1C=CC(=CC1)C=2C=C(C(=O)NC=3C=CC=4C=CC=CC4C3)C=5C=CC=CC5N2
IAL-MD0101	Not measurable	25.2	COC=1C=CC(=CC1)C=2C=C(C(=O)NC=3C=CC=C(C3)S(=O)(=O)N(C)C)C=4C=CC=CC4N2
IAL-MD0099	Not measurable	22.2	COC=1C=CC(=CC1)C=2C=C(C(=O)NCC(F)(F)F)C=3C=CC=CC3N2
IAL-MD0098	Not measurable	22.0	CCCNC(=O)C=1C=C(N=C2C=CC=CC12)C=3C=CC(OC)=CC3
IAL-MD0058	Not measurable	18.7	COC=1C=CC(=CC1)C=2C=C(C(=O)NC=3C=CC(=CC3)S(=O)(=O)C)C=4C=CC=CC4N2
IAL-MD0079	Not measurable	16.3	COC=1C=CC(=CC1)C=2C=C(C(=O)NC3=NN=CN3)C=4C=CC=CC4N2
IAL-MD0100	Not measurable	15.9	COC=1C=CC(=CC1)C=2C=C(C(=O)NC=3C=CC=NC3)C=4C=CC=CC4N2
IAL-MD0034	Not measurable	8.9	COC=1C=CC(=CC1)C=2C=C(C(=O)NC=3C=CC=C(OCC(=O)N)C3)C=4C=CC=CC4N2
IAL-MD0035	Not measurable	6.8	CCC(C)NC(=O)C=1C=C(N=C2C=CC=CC12)C=3C=CC(OC)=CC3
IAL-MD0089	Not measurable	5.7	OC(=O)C=1C=C(N=C2C=CC=CC12)C=3C=CC(OC(F)F)=CC3
**Singletons**			
IAL-MD0017	12.6	79.6	O=C(C1=C(N)C2=CC=C(C3=NC=CC=C3)N=C2S1)NC4CC4
IAL-MD0129	14.2	Not measurable due to compound precipitation	N#CC1=CC=CC=C1C2=CC=C(C3=NC=CN3CC4CN(C)CC4)O2
IAL-MD0127	38.0	66.5	O=C(C(O1)=C(C)C2=C1C(OCC)=C(OCC)C=C2)N[C@H]3[C@H](O)CNCC3.[H]Cl

Table S5. Hit compounds from FDA-approved compound library screen.

Compound library ID	Compound internal ID	NSP3 Mac1 inhibition at 50 μM (%)	Error (%)
Sennoside A	---	112.4	0.2
Ceftazidime	IAL-MD0001	107.9	0.1
Biotin	---	107.7	0.2
Ebselen	IAL-MD0015	107.7	0.0
Thimerosal	IAL-MD0006	107.3	0.1
Chlorophyllide Cu complex Na salt	---	106.7	0.4
Pyrithione zinc	---	100.7	0.1
Nadide	---	99.9	0.1
Carboplatin	IAL-MD0007	97.3	0.0
Cephalosporin C sodium	IAL-MD0228	89.8	2.2
Cisplatin	---	88.0	0.4
Methacycline hydrochloride	IAL-MD0008	86.8	0.3
Hexachlorophene	IAL-MD0009	85.5	4.1
Mitoxantrone hydrochloride	IAL-MD0010	81.9	4.2
Protoporphyrin IX	---	75.0	1.0
Ceftibuten	IAL-MD0002	71.6	0.4
Thioctic acid	IAL-MD0011	70.9	4.5
Zinc undecylenate	---	68.0	0.6
Bismuth subsalicylate	IAL-MD0012	66.0	21.9
Avobenzone	IAL-MD0003	61.8	0.2
Phenylmercuric acetate	---	58.1	4.7
Ethanolamine oleate	---	56.1	2.2
Oxantel pamoate	IAL-MD0013	55.9	0.0
Suramin hexasodium	---	55.8	0.7
Candicidin	---	50.1	1.6

Ethacridine lactate	IAL-MD0004	49.3	1.4
Sodium nitroprusside	---	46.7	0.4
Sulfanilate zinc	---	44.2	0.5
Aztreonam	IAL-MD0005	42.8	0.4
Cefepime hydrochloride	IAL-MD0229	42.1	1.8
